# Astragalin inhibits airway eotaxin-1 induction and epithelial apoptosis through modulating oxidative stress-responsive MAPK signaling

**DOI:** 10.1186/1471-2466-14-122

**Published:** 2014-07-29

**Authors:** In-Hee Cho, Ju-Hyun Gong, Min-Kyung Kang, Eun-Jung Lee, Jung Han Yoon Park, Sang-Jae Park, Young-Hee Kang

**Affiliations:** 1 Department of Food and Nutrition, Hallym University, Chuncheon, South Korea; 2 Medience Co., Ltd, Chuncheon, South Korea

**Keywords:** Asthma, Airway apoptosis, Astragalin, Eotaxin-1, Oxidative stress

## Abstract

**Background:**

Eotaxin proteins are a potential therapeutic target in treating the peribronchial eosinophilia associated with allergic airway diseases. Since inflammation is often associated with an increased generation of reactive oxygen species (ROS), oxidative stress is a mechanistically imperative factor in asthma. Astragalin (kaempferol-3-O-glucoside) is a flavonoid with anti-inflammatory activity and newly found in persimmon leaves and green tea seeds. This study elucidated that astragalin inhibited endotoxin-induced oxidative stress leading to eosinophilia and epithelial apoptosis in airways.

**Methods:**

Airway epithelial BEAS-2B cells were exposed to lipopolysaccharide (LPS) in the absence and presence of 1–20 μM astragalin. Western blot and immunocytochemical analyses were conducted to determine induction of target proteins. Cell and nuclear staining was also performed for ROS production and epithelial apoptosis.

**Results:**

When airway epithelial cells were exposed to 2 μg/ml LPS, astragalin nontoxic at ≤20 μM suppressed cellular induction of Toll-like receptor 4 (TLR4) and ROS production enhanced by LPS. Both LPS and H_2_O_2_ induced epithelial eotaxin-1 expression, which was blocked by astragalin. LPS activated and induced PLCγ1, PKCβ2, and NADPH oxidase subunits of p22^phox^ and p47^phox^ in epithelial cells and such activation and induction were demoted by astragalin or TLR4 inhibition antagonizing eotaxin-1 induction. H_2_O_2_-upregulated phosphorylation of JNK and p38 MAPK was dampened by adding astragalin to epithelial cells, while this compound enhanced epithelial activation of Akt and ERK. H_2_O_2_ and LPS promoted epithelial apoptosis concomitant with nuclear condensation or caspase-3 activation, which was blunted by astragalin.

**Conclusions:**

Astragalin ameliorated oxidative stress-associated epithelial eosinophilia and apoptosis through disturbing TLR4-PKCβ2-NADPH oxidase-responsive signaling. Therefore, astragalin may be a potent agent antagonizing endotoxin-induced oxidative stress leading to airway dysfunction and inflammation.

## Background

Bronchial epithelium is a key regulator of airway inflammation, airway wall remodeling and bronchial hyperresponsiveness in asthma, a complex immunologic and inflammatory disease [1]. There are secretion of cytokines and activation of inflammatory cells, including mast cells, T cells, eosinophils, and neutrophils [2-5]. There has been much circumstantial evidence implicating eosinophil infiltration into airway epithelium as one of major orchestrators in the pathophysiology of airway disorders [5]. The development of eosinophilic infiltration into the bronchial mucosa usually results in bronchial epithelial damage and airways hyperresponsiveness [6,7]. Accordingly, the bronchial epithelium is a target of inflammatory and physical insults as well as an effecter of ongoing airway inflammation. Accordingly, eosinophilic inflammation might be associated with the pathophysiology of acute exacerbations of airway diseases. Defining the mechanisms that control recruitment of eosinophils into the airway epithelium can propose potential targets for novel therapy. The eosinophil recruitment in experimental airway diseases entails the binding of eotaxin to C-C chemokine receptor type 3 expressed on eosinophils, basophils and Th2 cells [8].

Reactive oxygen species (ROS) can have detrimental effects on airway cells and tissues and hence oxidative stress contributes to the initiation and deterioration of inflammatory airway disorders such as asthma [9,10]. Several asthmatic mediators including chemokines and eosinophil granule proteins are potential stimuli of ROS production [11]. Some environmental factors linked to airway disorders such as air pollutants may also cause an extreme increase of ROS generation in the airway. In addition to inflammatory cells recruited to the asthmatic airway, constitutive airway cells such as epithelial cells are potential resources of ROS [12]. Numerous studies reported that the loss of antioxidant defenses was observed in airway disorders [13,14]. Accordingly, the modification of airway oxidative status may affect the pathological feature of airway disorders. It makes one take into account the necessity of antioxidants therapies [10,15].

Astragalin (kaempferol-3-O-glucoside), a newly found flavonoid from leaves of persimmon and green tea seeds, and its heptaacetate are known to have anti-tumor, anti-inflammatory and antioxidant activity [16,17]. Astragalin can improve survival during lethal endotoxemia by lipopolysaccharide (LPS) and attenuate inflammatory responses in a murine model of LPS-induced acute lung injury [18]. Additionally, this compound inhibits dermatitis development and IgE elevation in models of passive cutaneous anaphylaxis and atopic dermatitis NC/Nga mice [19]. However, the inhibitory effects of astragalin on asthmatic responses such as eotaxin-1 induction and epithelial apoptosis are not yet studied. Our recent study has demonstrated that kaempferol attenuates eosinophil infiltration and airway inflammation in allergic asthma through disturbing nuclear factor (NF)-κB signaling [20]. Another flavonoid fisetin with similar chemical structure exerts anti-asthma activity associated with reduction of Th2 responses and signaling suppression of NF-κB and downstream chemokines [21].

The cell membrane Toll-like receptor (TLR)4 recognizing LPS promotes inflammatory mechanisms including NF-κB [22]. The TLR4 activation activates eotaxin-1 protein necessary for the eosinophil recruitment to the inflammatory lesions [20]. It is deemed that TLR4 can trigger the crosstalk between eotaxin-1 induction and oxidative stress via redox-dependent mechanism (s). Based on the literature evidence that astragalin possesses antioxidant property and anti-allergic activity [18,19], this study investigated whether astragalin inhibited eotaxin-1 induction and apoptosis of airway epithelial BEAS-2B cells due to LPS-induced oxidative stress. Furthermore, this study elucidated whether astragalin encumbered airway eosinophilia and epithelial apoptosis through disturbing TLR4-NADPH oxidase pathway responsive to LPS.

## Methods

### Chemicals

M199, human epidermal growth factor (EGF), hydrocortisone, gelatin, human insulin, apotransferrin, LPS and H_2_O_2_ were obtained from the Sigma-Aldrich Chemical (St. Louis, MO), as were all other reagents, unless specifically stated elsewhere. Fetal bovine serum (FBS), penicillin-streptomycin and trypsin-EDTA were purchased from the Lonza (Walkersville, MD). The human bronchial airway epithelial cell line BEAS-2B cells were provided by the American Type Culture Collection (Manassas, VA). 3-(4, 5-Dimetylthiazol-yl)-diphenyl tetrazolium bromide (MTT) was obtained from Duchefa Biochemie (Haarlem, Netherlands). Human eotaxin-1 (CCL11) antibody was purchased from R&D systems (Minneapolis, MN) and human β-actin antibody obtained from Sigma-Aldrich Chemicals. Antibodies against human TLR4, human p22^phox^ and human p47^phox^ were purchased from the Santa Cruz Biotechnology (Santa Cruz, CA). Antibodies against human phospho-PKC(pan)β2 (Ser660), human PLCγ1, human phospho-ERK (p44/42 MAPK, Thr202/Tyr204), human phospho-Akt, human phospho-SAPK/JNK (Thr183/Tyr185), human phospho-p38 MAPK (Thr180/Tyr182) and human cleaved caspase-3 were provided by Cell Signaling Technology (Beverly, MA). Horseradish peroxidase-conjugated goat anti-rabbit IgG, donkey anti-goat IgG and goat anti-mouse IgG were purchased from Jackson ImmunoResearch Laboratories (West Grove, PA). 4',6-Diamidino-2-phenylindole (DAPI) and 2′-7′-dichlorodihydrofluorescein diacetate (DCF-DA) was obtained from Santa Cruz Biotechnology.

Astragalin was dissolved in dimethyl sulfoxide (DMSO) for live culture with cells; a final culture concentration of DMSO was ≤0.5%. The present study was approved by the Hallym University Institutional Review Board and Committee on Animal Experimentation (hallym R2014-6).

### BEAS-2B cell culture and viability

The human lung bronchus epithelial BEAS-2B cells were cultured in 25 mM HEPES-buffered M199 containing 10% FBS, 2 mM L-glutamine, 100 U/ml penicillin, 100 μg/ml streptomycin supplemented with 2.5 μg/ml insulin, 361 ng/ml hydrocortisone, 2.5 μg/ml apo-transferrin and 20 ng/ml EGF. BEAS-2B cells were sustained in 90-95% confluence at 37°C in an atmosphere of 5% CO_2_. LPS at the concentration of 2 μg/ml was applied to BEAS-2B cells for up to 8 h to induce target gene protein expression and ROS production [20]. In another set of experiments, cells were pretreated for 30 min with 1–20 μM astragalin and then exposed to 20 μM H_2_O_2_ for 24 h. Non-H_2_O_2_-treated cells were also incubated under the same conditions as those used for the H_2_O_2_ protocols. Cells were washed and resupplied with a fresh medium containing 1–20 μM astragalin.

**Figure 1:**
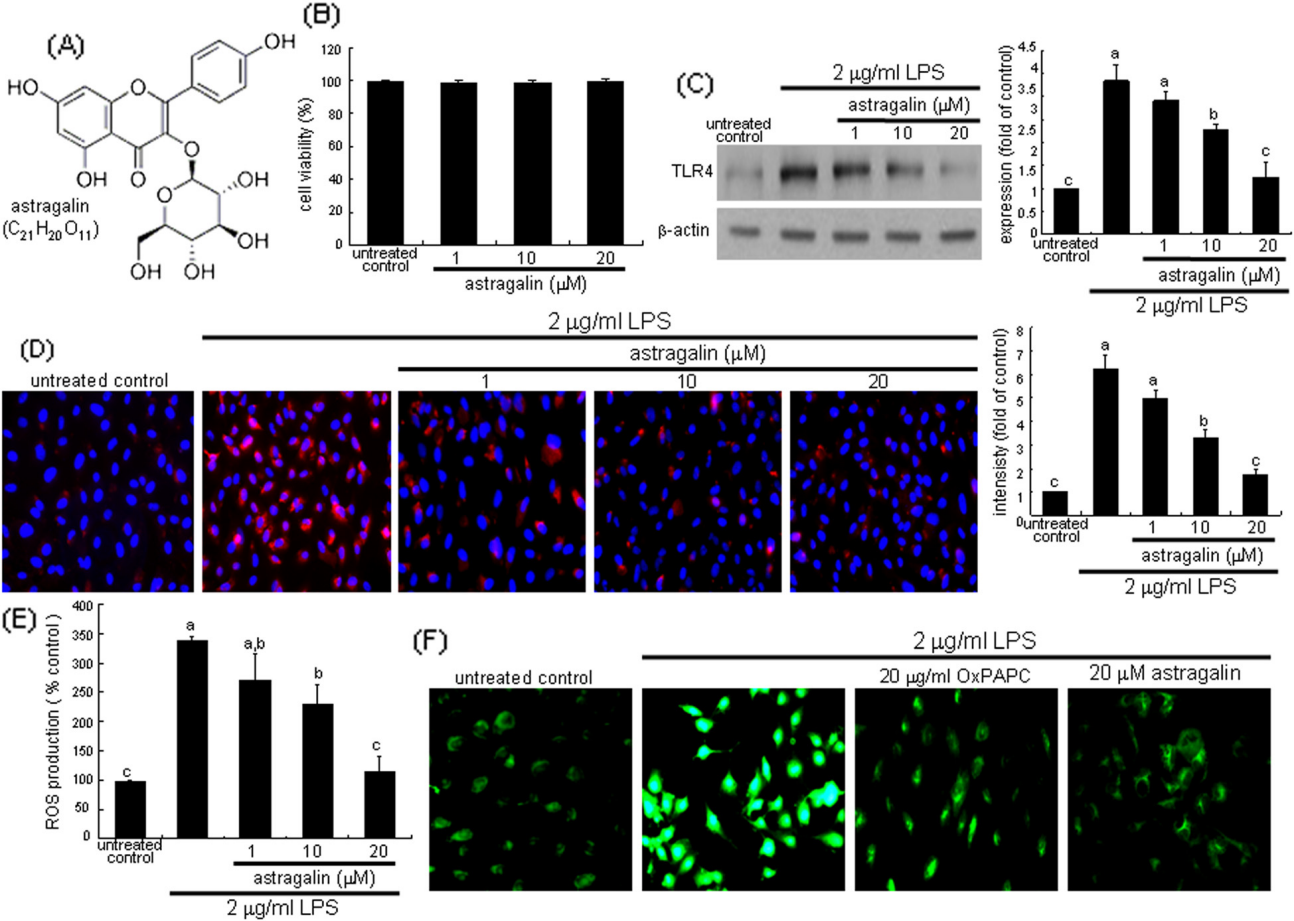
**Chemical structure of astragalin (A) and viability of BEAS-2B cells treated with 1–20** **μM astragalin for 24 h (B).** Cell viability was measured by MTT assay and viability data are mean ± SEM (n = 5, cell viability of untreated controls = 100%). Inhibitory effects of astragalin on TLR4 expression in LPS-exposed BEAS-2B cells **(C andD)**. BEAS-2B cells were cultured with 2 μg/ml LPS in the absence and presence of 1–20 μM astragalin for 8 h. Cell lysates were prepared for Western blotting with a primary antibody against TLR4 **(C)**. β-Actin protein was used as an internal control. The bar graphs (means ± SEM, n = 3) represent quantitative results of the upper bands obtained from a densitometer. Fluorescent images for the TLR4 induction were obtained by binding with a Cy3-conjugated IgG and by counterstaining with DAPI **(D)**. For the measurement of ROS formation, cells were incubated with 10 μM DCF-DA. At the end of DCF-DA incubation, cells were lysed with NaOH and fluorescence was measured **(E)**. Fluorescent images **(F)** were obtained with a fluorescence microscopy. Image magnification: x200. Means in bar graphs (mean ± SEM, n = 3) not sharing a common superscript refer to significant different at *P* < 0.05.

The cytotoxicity of astragailn or H_2_O_2_ was determined using MTT assay after culture of BEAS-2B cells. BEAS-2B cells were incubated in a fresh medium containing 1 mg/ml MTT for 3 h at 37°C. The purple formazan product was dissolved in 0.5 ml isopropanol with gentle shaking. Absorbance of formed formazan was measured at λ = 570 nm using a microplate reader (Bio-Rad Model 550, Hercules, CA). In the current study astragalin *per se* did not induce toxicity of BEAS-2B cells at 1–20 μM (Figure 1B).

### Western blot analysis

Whole BEAS-2B cell lysates were prepared in 1 mM Tris–HCl (pH 6.8) lysis buffer containing 10% SDS, 1% glycerophosphate, 0.1 mM Na_3_VO_4_, 0.5 mM NaF and protease inhibitor cocktail. Each cell lysate containing equal amounts of proteins was electrophoresed on 8-15% SDS-PAGE and transferred onto a nitrocellulose membrane. Blocking a non-specific binding was performed using either 3% fatty acid-free bovine serum albumin or 5% non-fat dry milk for 3 h. The membrane was incubated overnight at 4°C with a specific primary antibody of TLR4, eotaxin-1, PLCγ1, phospho-PKCβ2, NADPH oxidases, phosphorylated MAPK of JNK, p38, ERK and Akt or cleaved caspase-3. The membrane was then applied to a secondary antibody of goat anti-rabbit IgG or goat anti-mouse IgG conjugated to horseradish peroxidase for 1 h. Following another triple washing, each target protein level was determined using the Supersignal West Pico Chemiluminescence detection reagents (Pierce Biotechnology, Rockford, IL) and the Agfa medical X-ray film blue (Agfa HealthCare NV, Mortsel, Belgium). Incubation with mouse anti-human β-actin was conducted for the comparative control.

### Immunocytochemical analysis

Immunofluorescent cytochemical staining for monolayer cells of BEAS-2B cells grown on glass coverslips was performed using human TLR4 antibody and Cy3-conjugated anti-rabbit IgG. BEAS-2B cells were fixed with 4% formaldehyde for 15 min and permeated with 0.1% Triton X-100 for 2 min on ice. Cells were blocked using a 4% FBS for 1 h, and anti-human TLR4 was applied to cells. Triple washing was followed and incubation with Cy3-conjugated goat anti-rabbit IgG was achieved for 1 h. Nuclear staining was carried out with 4 mg/ml DAPI. Each slide was mounted in VectaMount mounting medium (Vector Laboratories, Burlingame, CA). Images of each slide were taken using an optical microscope system (Axiomager, Zeiss, Oberkochen, Germany). The TLR4 protein level was quantified with an image analysis program from the microscope system.

### Intracellular oxidant generation

Intracellular ROS production was detected by loading DCF-DA hydrolyzed and oxidized by ROS to a fluorescent compound DCF. BEAS-2B cells were treated with 2 μg/ml LPS in the presence of 1–20 μM astragalin for 8 h, and were incubated in DCF-DA for 30 min. After twice washes with phosphate buffered saline (PBS), BEAS-2B cells were lysed in 0.1 M NaOH. The equal amounts of cell lysates were transferred to 96-black well plates and the fluorescence was measured at λ = 485 nm excitation and λ = 538 nm emission. After we completed dye loading at 37°C, the cells were rinsed twice with PBS, and the cultures were photographed with a fluorescence microscope.

### Nuclear staining

After treating 1–20 μM astragalin and 20 μM H_2_O_2_ to BEAS-2B cells grown on a glass-chamber slide, a brief washing with PBS-0.2% Tween 20 was conducted. BEAS-2B cells were fixed with 4% formaldehyde for 15 min. After blocking cells with a 4% FBS for 1 h, cells were stained with 4 mg/ml DAPI to visualize nuclear condensation and fragmentation under fluorescence microscopy. Images of each slide were taken using an optical microscope system (Axiomager).

### Statistical analysis

The results were expressed as mean ± SEM in each experiment. Statistical analyses were performed using Statistical Analysis Systems statistical software package (SAS Institute, Cary, NC). Significance was determined by one-way analysis of variance, followed by Duncan range test for multiple comparisons. Differences were considered significant at P < 0.05.

## Results

### Suppression of LPS-promoted TLR4 induction by astragalin

The TLR4 expression was very weak in untreated quiescent cells, whereas the TLR4 induction was greatly elevated in 2 μg/ml LPS-exposed BEAS-2B cells (Figure 1C). When epithelial cells were supplemented with ≥10 μM astragalin for 8 h, the TLR4 induction was significantly inhibited. Furthermore, the immunocytochemical analysis examined the localization of cellular TLR4 in LPS-stimulated BEAS-2B cells, evaluated by fluorescent microscopy using a specific TLR4 antibody. There was lack of cell membrane staining in the untreated cells (Figure 1D). However, in LPS-stimulated BEAS-2B cells for 8 h heavy pinkish staining was observed outside blue colored-nuclei. The pinkish staining for the TLR4 induction was substantially and dose-dependently diminished in cells supplemented with astragalin (Figure 1D).

### Astragalin inhibition of eotaxin-1 induction by oxidative stress

We determined that in LPS-exposed cells astragalin influenced intracellular oxidant formation, as evidenced by oxidation of DCF-DA. It was found that LPS highly accelerated ROS production by 3.5-fold from BEAS-2B cells (Figure 1E). The enhanced ROS production was dose-dependently suppressed by supplementing astragalin to epithelial cells. Figure 1F shows the expected weak staining in the LPS-free control. The LPS-alone-exposed cells showed heavy fluorescence, indicative of marked oxidant generation. The cells exposed to LPS in the presence of 20 μg/ml OxPAPC, a TLR2/4 signaling inhibitor [23], revealed no increase in DCF fluorescence, indicating that astragalin blocked an accumulation of intracellular oxidants in epithelial cells due to LPS through disturbing TLR signaling (Figure 1F).

**Figure 2:**
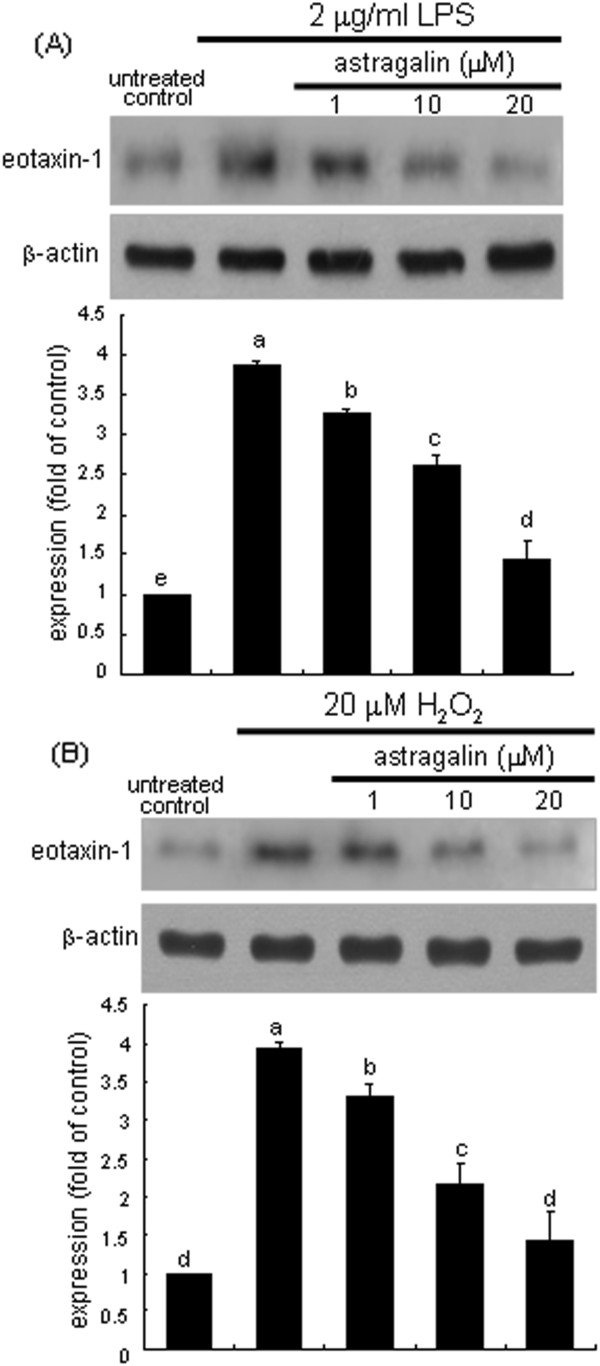
**Inhibition of eotaxin-1 expression by astragalin in LPS- or H**_**2**_**O**_**2**_**-exposed BEAS-2B cells.** After BEAS-2B cell culture protocols with 2 μg/ml LPS for 8 h **(A)** and or 20 μM H_2_O_2_ for 24 h **(B)**, cell lysates were prepared for Western blotting with anti-eotaxin-1. β-Actin protein was used as an internal control. The bar graphs (mean ± SEM, n = 3-4) represent quantitative results of the upper bands obtained from a densitometer. Means not sharing a common superscript refer to significant different at *P* < 0.05.

This study investigated whether oxidative stress was involved in the induction of eotaxin-1 protein in LPS-experienced airway epithelial cells, which was inhibited by treating astragalin. Eotaxin-1 expression was markedly elevated in LPS-elicited BEAS-2B cells (Figure 2A). In contrast, astragalin nontoxic at 1–20 μM attenuated the eotaxin-1 induction in a dose-dependent manner. These observation data imply that LPS-induced oxidants appeared to be responsible for eotaxin-1 induction. Similarly, 20 μM H_2_O_2_ greatly induced epithelial eotaxin-1 from airway cells, which was reversed by treating 1–20 μM astragalin (Figure 2B). Accordingly, oxidative stress may be involved in LPS-induction of airway epithelial eotaxin-1.

### Blockade of PLCγ1-PKCβ2-NADPH oxidase pathway by astragalin

**Figure 3:**
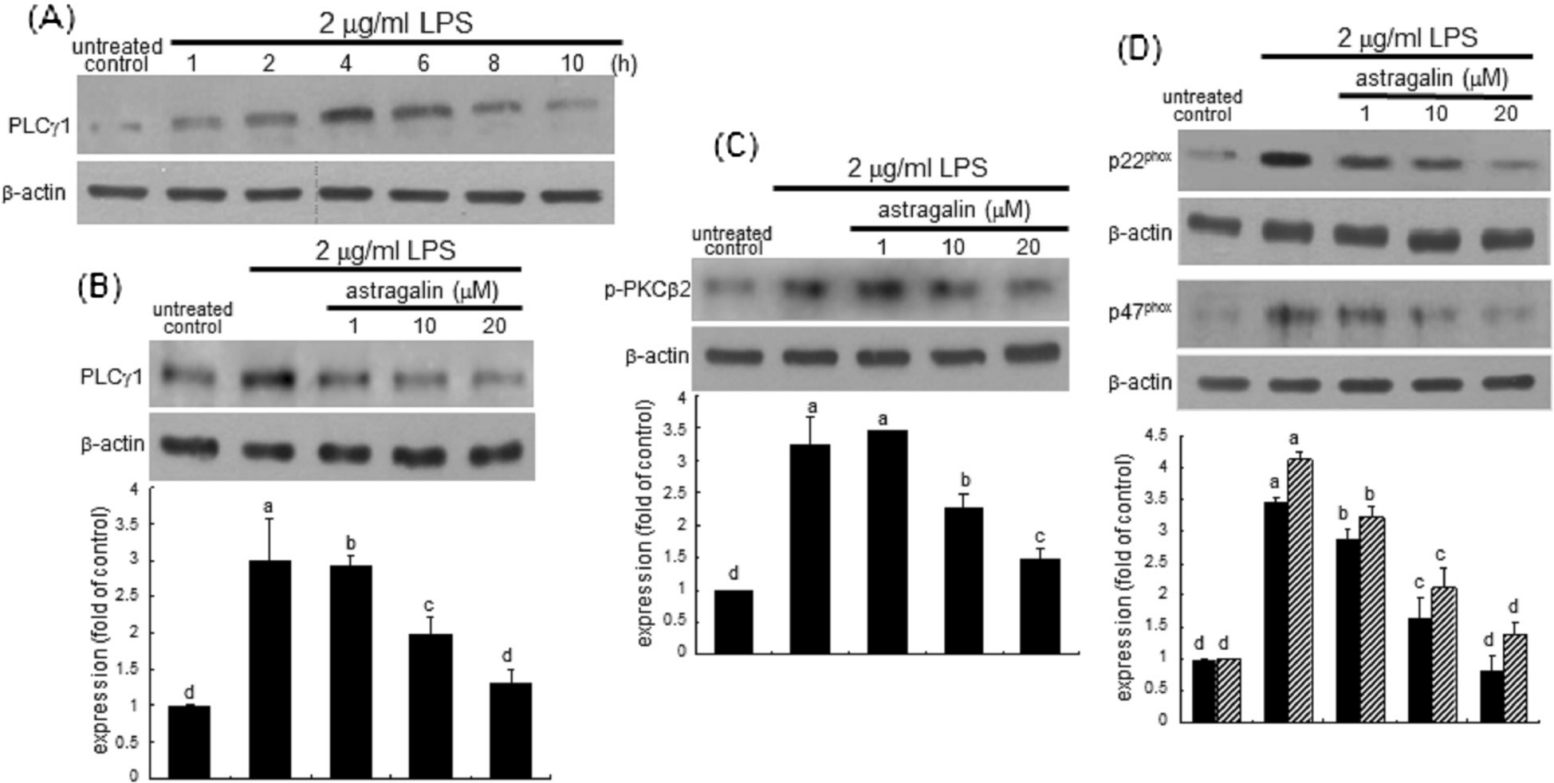
**Time course response of PLCγ1 induction (A) and astragalin suppression of LPS-induced expression or activation of PLCγ1 (B), PKCβ2 (C), NADPH oxidases (D) by astragalin in BEAS-2B cells.** Cells were treated with 1–20 μM astragalin and then stimulated with 2 μg/ml LPS for 8 h. Cell lysates were prepared for Western blot analysis with a primary antibody against PLCγ1, phospho-PKCβ2, p22^phox^, and p47^phox^. β-Actin protein was used as an internal control. The bar graphs (mean ± SEM, n = 3-5) represent quantitative results of the upper bands obtained from a densitometer Means not sharing a common superscript refer to significant different at *P* < 0.05.

This study elucidated whether LPS induced cellular signal transduction of PLCγ1-PKCβ2-NADPH oxidases through stimulating TLR4 signaling, which was disturbed by astragalin. The stimulation of epithelial cells by LPS highly activated PLCγ1 in a temporal manner with its peak expression at 4 h (Figure 3A). Adding astragalin to LPS-exposed BEAS-2B cells significantly inhibited the activation of PLCγ1, relative to that of LPS-alone-exposed cells (Figure 3B). Additionally, astragalin suppressed the phosphorylation of PKCβ2 elicited by stimulating airway epithelial BEAS-2B cells with 2 μg/ml LPS (Figure 3C). It was also found that LPS significantly enhanced cellular expression of p22^phox^ and p47^phox^ and astragalin encumbered such increased expression in a dose-dependent manner (Figure 3D). This indicates that astragalin ameliorated LPS-mediated eotaxin-1 via disturbing PLCγ1-PKCβ2-NADPH oxidase signaling.

**Figure 4:**
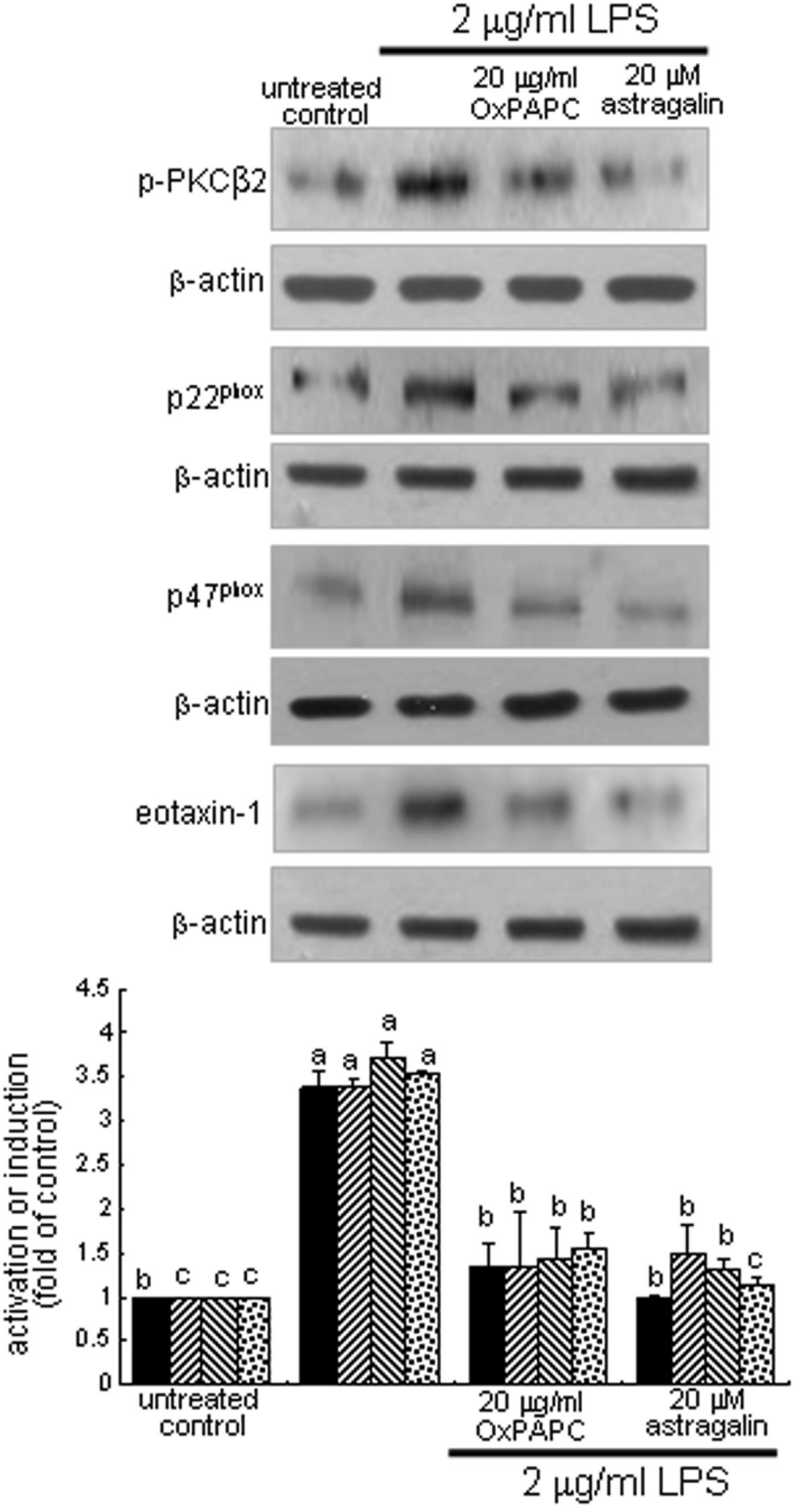
**Suppression of LPS induction of NADPH oxidases by TLR4 blockade in BEAS-2B cells. Cells were treated with 20** **μM astragalin or 20** **μg/ml OxPAPC and then stimulated with 2** **μg/ml LPS for 8 h.** Cell lysates were prepared for Western blot analysis with a primary antibody against phospho-PKCβ2, p22^phox^, p47^phox^, and eotaxin-1. β-Actin protein was used as an internal control. The bar graphs (mean ± SEM, n = 3-4) represent quantitative results of the upper bands obtained from a densitometer. Means not sharing a common superscript refer to significant different at *P* < 0.05.

This study attempted to confirm that the TLR4 stimulation elicited by LPS was linked to the eotaxin-1 induction and the PLCγ1-NADPH oxidase signaling. The LPS-up-regulated PKCβ2 activation was dampened by non-toxic OxPAPC at 20 μg/ml (Figure 4). Similar inhibition was observed with 20 μM astragalin. In addition, cellular expression of p22^phox^ and p47^phox^ up-regulated by LPS was abolished by the TLR4 blockade of BEAS-2B cells, as with eotaxin-1 induction by 2 μg/ml LPS (Figure 4). Consequently, it was deemed that the ROS production by LPS via its activation of TLR4-PKCβ2-NADPH oxidase signaling induced bronchial eoaxin-1, which was deterred by treating astragalin to epithelial cells.

### Inhibitory effect of astragalin on ROS-triggered apoptosis and MAPK activation

**Figure 5:**
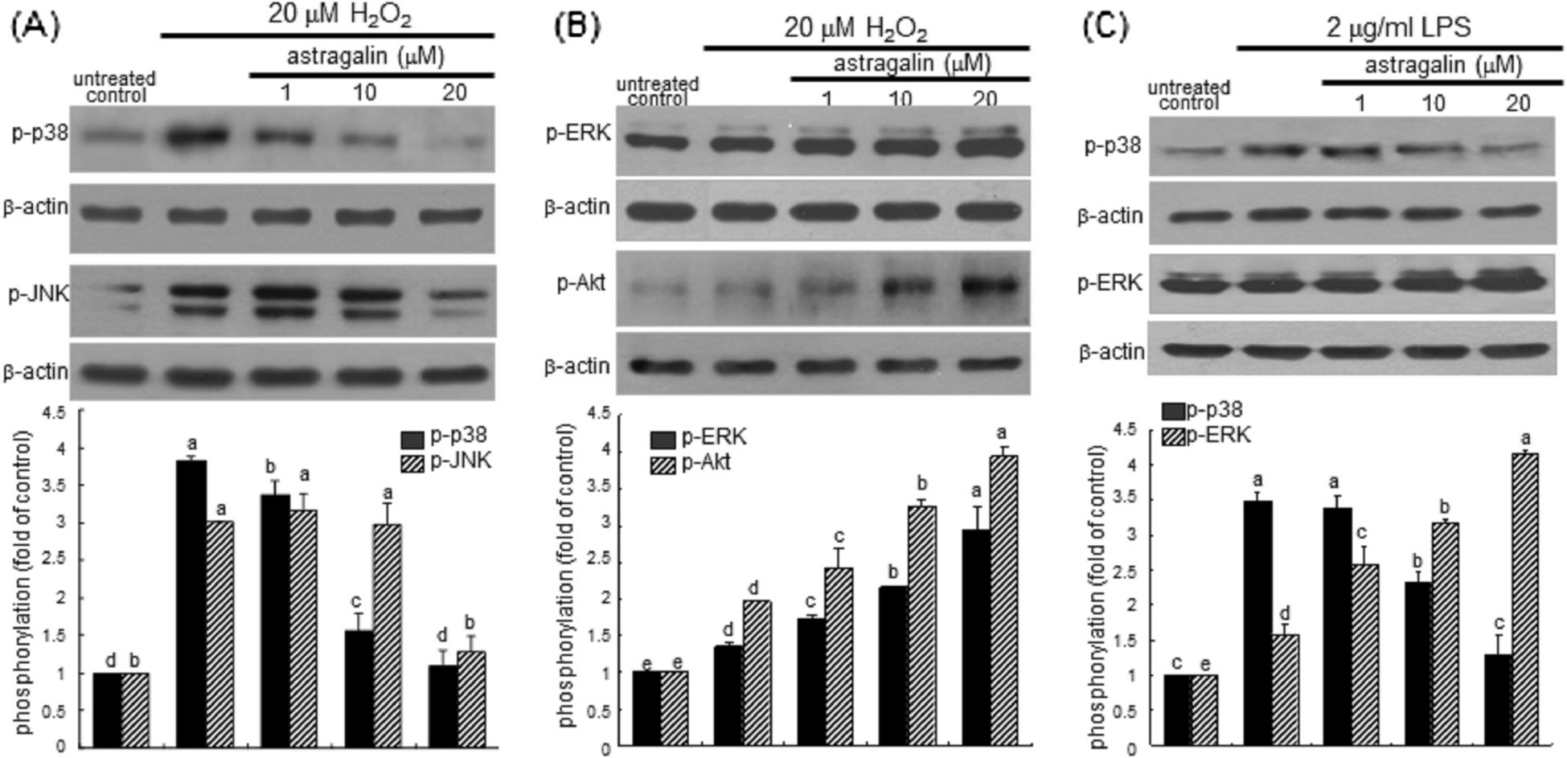
**Effects of astragalin on H**_**2**_**O**_**2**_**(A and B)- or LPS (C)-induced activation of MAPK in BEAS-2B cells.** Cells were exposed to 20 μM H_2_O_2_ for 24 h or 2 μg/ml LPS for 8 h in the absence and presence of 1–20 μM astragalin. Cell lysates were prepared for Western blot analysis with a primary antibody against phospho-ERK, phospho-Akt, phospho-p38, and phospho-JNK. β-Actin protein was used as an internal control. The bar graphs (mean ± SEM, n = 3-4) represent quantitative results of the upper bands obtained from a densitometer. Means not sharing a common superscript refer to significant different at *P* < 0.05.

LPS produced ROS in epithelial BEAS-2B cells through stimulating TLR4-NADPH oxidase signaling (Figure 1E and Figure 4). When BEAS-2B cells were incubated with the ROS buster H_2_O_2_ at 20 μM, the phosphorylation of p38 MAPK and JNK was highly enhanced (Figure 5A). However, the activation of p38 MAPK and JNK was dose-dependently suppressed in H_2_O_2_-exposed BEAS-2B cells in the presence of ≥1 μM astragalin (Figure 5A). Furthermore, this study examined the activation of other MAPK proteins of ERK and Akt possibly involved in the airway tissue damage. H_2_O_2_*per se* minimally but significantly up-regulated epithelial phosphorylation of ERK and Akt (Figure 5B). When astragalin was supplemented to H_2_O_2_-exposed BEAS-2B cells, the activation of ERK and Akt was further dose-dependently enhanced. Accordingly, the ROS producer LPS like H_2_O_2_ affected MAPK signaling pathway(s) of p38 and ERK in epithelial cells (Figure 5C).

**Figure 6:**
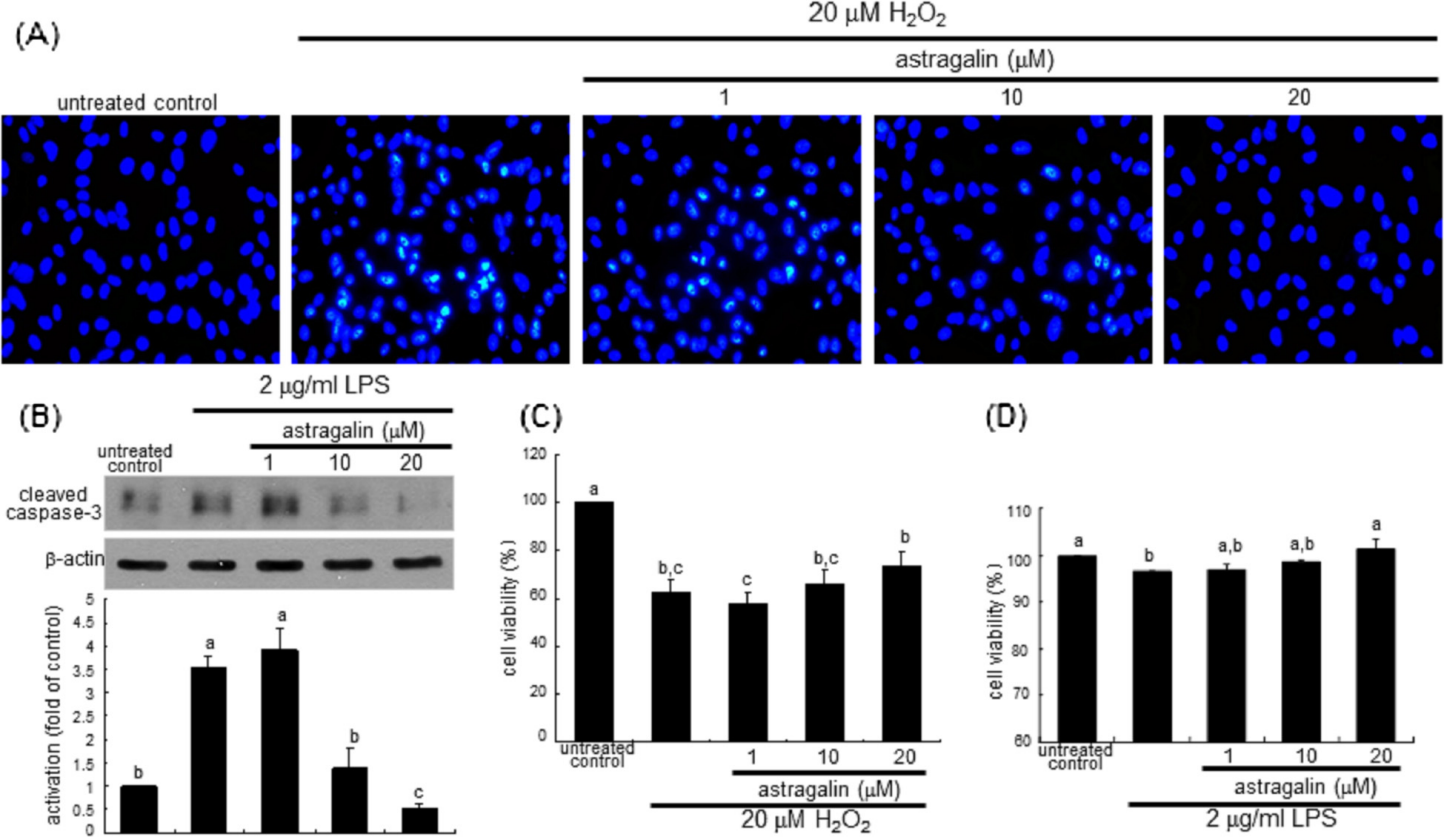
**Improvement of nuclear condensation and fragmentation (A), inhibition of caspase-3 activation (B) and enhancement of cell viability (C and D) by astragalin in H**_**2**_**O**_**2**_**-exposed or LPS-exposed BEAS-2B cells.** Cells were exposed to 20 μM H_2_O_2_ for 24 h or 2 μg/ml LPS for 8 h in the absence and presence of 1–20 μM astragalin, and DAPI staining or MTT assay was subsequently performed. Nuclear condensation and fragmentation were observed with fluorescence microscopy. Representative microphotographs were obtained from three independent experiments. The MTT data for viability are mean ± SEM (n = 5, cell viability of untreated controls = 100%). Cell lysates were prepared for Western blot analysis with a primary antibody against cleaved caspase-3. β-Actin protein was used as an internal control. The bar graphs (mean ± SEM, n = 3-4) represent quantitative results of the upper bands obtained from a densitometer. Means not sharing a common superscript refer to significant different at *P* < 0.05.

Shedding of bronchial epithelial cells is characterized by loss of the normal bronchial pseudostratified epithelium [24]. It was elucidated that MAPK signaling pathway(s) was responsible for oxidative stress-associated airway epithelial damage. H_2_O_2_ elicited nuclear condensation and fragmentation of BEAS-2B cells, evidenced by DAPI staining (Figure 6A). In contrast, astragalin inhibited such nuclear changes, indicating that astragalin can also dampen epithelial apoptosis by oxidative stress triggered by LPS. Consistently, the apoptotic caspase-3 was activated in LPS-experienced epithelial cells, which was attenuated by astragalin (Figure 6B). This finding demonstrates that astragalin inhibited LPS-elicited airway epithelial apoptosis through disturbing the ROS activation of p38 MAPK and promoting the activation of ERK (Figure 5C). Finally, 20 μM astragalin increased the viability of H_2_O_2_-exposed BEAS-2B cells by ≈ 15% (Figure 6C). Also, astragalin appeared to demote the susceptibility to apoptosis of LPS-exposed bronchial epithelial cells (Figure 6D).

## Discussion and conclusion

Six major findings were extracted from this study. 1) When airway epithelial cells were exposed to 2 μg/ml LPS, bronchial epithelial cell TLR4 was markedly induced. This induction was dose-dependently attenuated by astragalin at 1–20 μM. 2) There was a marked increase in ROS production of epithelial cells by LPS, which was suppressed by adding astragalin. 3) Both the oxidant H_2_O_2_ and LPS increased cellular expression of eotaxin-1 of airway epithelial BEAS-2B cells. Such induction was significantly suppressed by the treatment of epithelial cells with astragalin non-toxic at ≤20 μM. 4) Astragalin blunted the LPS activation and induction of PLCγ1, PKCβ2 and NADPH oxidase subunits of p22^phox^ and p47^phox^ through disturbing TLR4 signaling pertaining to epithelial induction of eotaxin-1. 5) H_2_O_2_ promoted epithelial apoptosis concomitant with rapid phosphorylation of JNK and p38, which was reversed by astragalin. In contrast, the activation of Akt and ERK was up-regulated due to the presence of astragalin. 6) LPS activated caspase-3 in epithelial cells, indicating that LPS induced epithelial apoptosis. Astragalin deterred such apoptosis and enhanced epithelial cell survival. Therefore, astragalin can be effective in ameliorating asthmatic airway diseases through modulating oxidative stress-responsive signaling pathway linked to LPS-TLR4 signaling. It cannot be ruled out that there may be a direct binding of LPS to astragalin in triggering LPS-TLR4 signaling.

Eosinophil infiltration into bronchial epithelium occurs in the episode of asthma and results in airway epithelial damage and hyperresponsiveness [5-7]. Eosinophils release highly- cytotoxic granular proteins eliciting detrimental tissue damage and severe inflammation [25]. Accordingly, the blockade of eotaxin-1-induced eosinophil recruitment may provide a novel therapeutic strategy for airway disorders. Soy isoflavone dramatically inhibits ovalbumin-induced eosinophil infiltration of lung tissues and mucus production in a murine model of asthma [26]. Soy isoflavone may be a novel means for the treatment of airway inflammatory disease. Astragalin found in persimmon leaves and green tea seeds, and its heptaacetate have various bioactive actions including anti-tumor, anti-inflammatory and antioxidant activities [16,17]. This study revealed that astragalin suppressed the eotaxin-1 induction possibly linked to TLR4 signaling by LPS. Accordingly, this compound may block eotaxin-1-associated eosinophilia in endotoxin-induced airway disorders. Other investigation has shown that astragalin inhibits dermatitis development and IgE elevation in models of passive cutaneous anaphylaxis and atopic dermatitis NC/Nga mice [19]. However, the mechanism (s) by which the inhibitory effects of astragalin on asthmatic responses such as eotaxin-1 induction are not yet defined. The present study showed that the eotaxin-1 induction by LPS was linked to oxidative stress due to ROS production and that astragalin suppressed oxidant-induced eotaxin-1 expression with a concurrent reduction of ROS production. One can assume that this inhibitory effect of astragalin was attributed to its scavenging activity of ROS or its direct binding between astragalin and H_2_O_2_. The antioxidant flavonoid naringenin attenuates mucous hypersecretion through modulating ROS production in human airway epithelial cells [27]. In addition, tea epigallocatechin-3-gallate diminished ROS generation in bronchoalveolar lavage fluid by toluene diisocyanate inhalation and protected airway inflammation in a murine model of asthma [28]. This study did not examine the inhibition of ROS generation by astragalin in a murine asthma model. Nevertheless, it can be assumed that the antioxidant astragalin can suppress *in vivo* airway disorders.

Inflammatory cells recruited to the asthmatic airway have an exceptional capability for producing ROS [29]. Mediators secreted in the asthmatic airway are potential stimuli of ROS production [11]. Also, the constitutive airway cells such as epithelial cells are potential resources of ROS [12]. Activated inflammatory cells such as eosinophils, neutrophils, monocytes, and macrophages can generate superoxides via the NADPH oxidase-dependent complex. This study found that LPS highly enhanced cellular expression of NADPH oxidase subunits of p22^phox^ and p47^phox^, which was blunted by astragalin concomitantly with the dose-dependent inhibition of PKCβ2 activation. It was also shown that the cell membrane-associated TLR4 signaling triggered the crosstalk between eotaxin-1 induction and oxidative stress via modulating redox-dependent mechanism (s). Accordingly, astragalin appeared to suppress oxidant-induced airway eosinophilia through disturbing TLR4-PKCβ2-NADPH oxidase signaling responsive to LPS in the airway epithelial cell system. Naringenin minimizes ROS production-associated mucous production during airway inflammation by inhibiting NF-κB activity via EGF receptor-phosphatidylinositol 3-kinase-Akt/ERK signaling pathway [27]. Some reports show that increased oxidative stress and decreased levels of antioxidants were observed in asthma [13,14]. However, the endogenous antioxidant ability limits the extent of cellular injury from oxidants during allergic insults.

**Figure 7:**
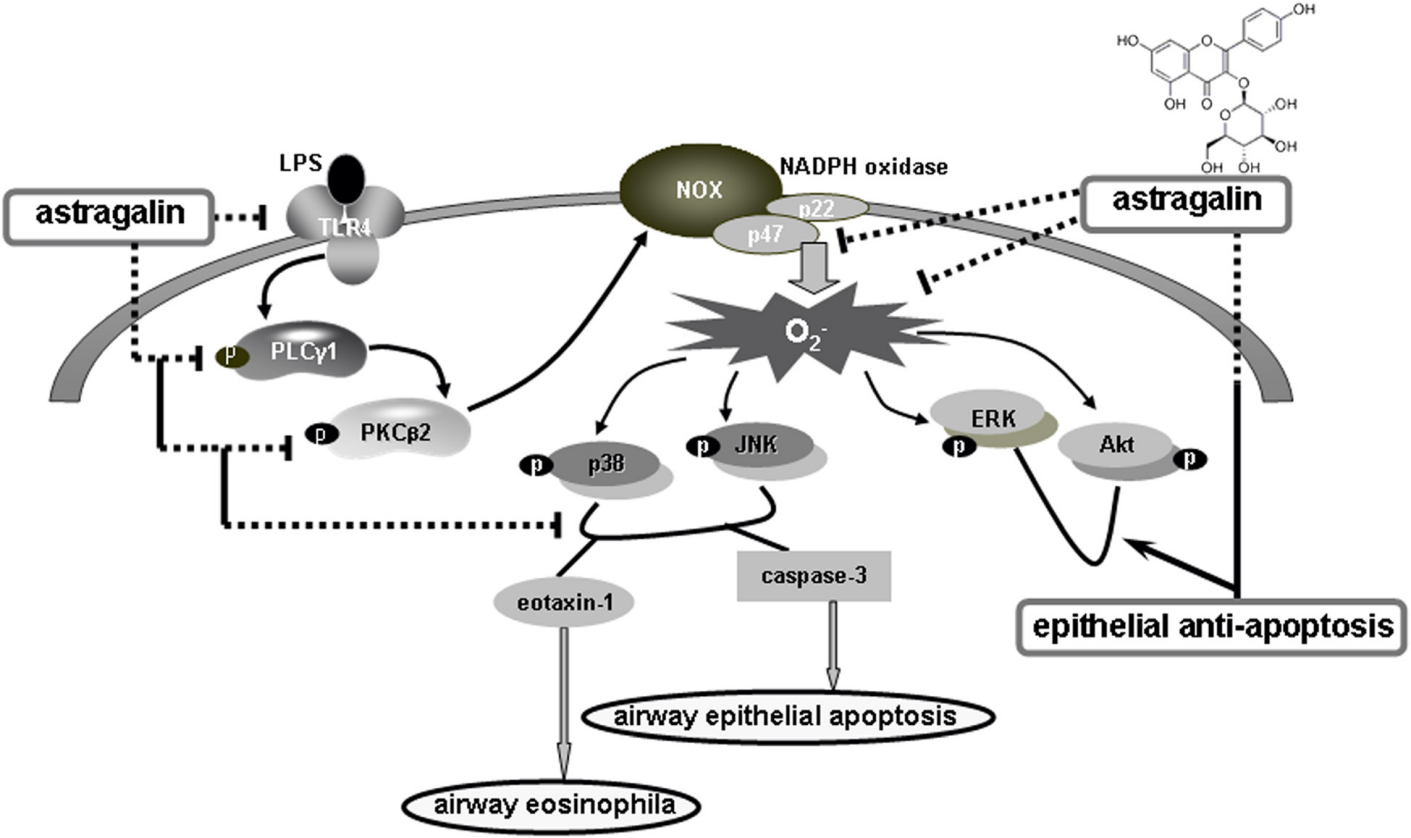
**Schematic diagram showing anti-eosinophilic and anti-apoptotic features of astragalin and its mechanistic actions in endotoxin-induced airway disorders.** As depicted, astragalin inhibits the direct asthmatic signaling cascades induced by LPS. Arrows indicate activation or induction; ⊥ indicates inhibition or blockade.

Airway epithelial cells are easily susceptible to inhaled noxious agents and relatively refractory to apoptotic stimuli. It has been reported that epithelial apoptosis of the chronically inflamed airway can be highly increased [30]. Survival mechanism (s) need in place to maintain the integrity of the epithelial barrier that is exposed to agents such as ROS and death receptor ligands secreted by immune cells during inflammation. Thus, there is a necessity to develop strategies to minimize ongoing damages to the airway epithelium and consequent airway remodeling. Targeting major apoptosis-manipulating factors in the inflamed airway epithelium would be one of such strategies. This study showed that the oxidant H_2_O_2_ promoted epithelial apoptosis by up-regulating NADPH oxidases responsible for the activation of JNK and p38 MAPK, which was blocked by astragalin. One can think that the specific inhibition of JNK and p38 MAPK signaling would be necessary for their involvement in epithelial apoptosis. The survival mechanisms by which astragalin inhibited epithelial apoptosis due to ROS entailed the enhanced activation of ERK signaling. The methoxyflavonoid nobiletin inhibits eosinophilic airway inflammation by promoting eosinophil apoptosis by enhancing Fas transcription in asthmatic rats [31]. Natural polyphenols hold promise as anti-asthmatic agents capable of influencing multiple signaling pathways and immune cell functions [32].In summary, this study investigated the potential of astragalin as a target antagonizing endotoxin- or oxidative stress-associated airway eosinophilia and epithelial apoptosis. Nontoxic astragalin suppressed LPS-induced ROS production and eotaxin-1 expression in epithelial cells. The LPS induction of eotaxin-1 was linked to oxidative stress through triggering the signaling pathway of TLR4-PKCγ1-PKCβ2-NADPH oxidases disturbed by astragalin. Additionally, astragalin attenuated endotoxin-instigated epithelial apoptosis through manipulating oxidative stress-elicited MAPK signaling in airway epithelial cells. Therefore, astragalin might be effective in ameliorating oxidative stress-associated epithelial eosinophilia and apoptosis through disturbing TLR4-PKCβ2-NADPH oxidase-responsive signaling in a cellular model of airway disorder (Figure 7). Although astragalin may serve as a modulator against asthma in vitro, its dietary in vivo role as an anti-asthmatic agent remains unclear.

## Abbreviations

ERK: Extracellular signal-regulated kinase; JNK: Jun N-terminal kinase; LPS: Lipopolysaccharide; MAPK: Mitogen-activated protein kinase; NF-κB: Nuclear factor-κB; PKC: Protein kinase; ROS: Reactive oxygen species; TLR: Toll-like receptor.

## Competing interests

I-H Cho, J-H Gong, M-K Kang, E-J Lee, J H Y Park, S-J Park, and Y-H Kang no conflicts of interest.

## Authors’ contributions

IHC, JHG, and YHK designed research; IHC, JHG, EJL and SJP conducted research; IHC, JHG, and MKK analyzed data; JHYP and YHK wrote the paper. YHK had primary responsibility for final content. All authors read and approved the final manuscript.

## Pre-publication history

The pre-publication history for this paper can be accessed here:

http://www.biomedcentral.com/1471-2466/14/122/prepub
